# Biopsy-Confirmed Cerebral Amyloid Angiopathy-Related Inflammation With Absence of Cerebral Microbleeds on T2* and Detectable Only by Susceptibility-Weighted Imaging

**DOI:** 10.7759/cureus.95804

**Published:** 2025-10-31

**Authors:** Takeshi Araki, Hisatsugu Tachibana, Yoshihiro Ikura, Tatsuo Matsushita

**Affiliations:** 1 Neurology, Takatsuki General Hospital, Osaka, JPN; 2 Pathology, Takatsuki General Hospital, Osaka, JPN

**Keywords:** amyloid-beta-related angiitis, cerebral amyloid angiopathy-related inflammation, cerebral microbleeds, leptomeningeal enhancement, susceptibility-weighted imaging

## Abstract

Cerebral amyloid angiopathy-related inflammation (CAA-ri) is a rare but treatable inflammatory condition in which there is amyloid-beta deposition in cortical and leptomeningeal vessels. Patients with CAA-ri often present with a high number of cerebral microbleeds (CMBs). Susceptibility-weighted imaging (SWI) is more sensitive than T2* for detecting these CMBs. However, SWI is not currently mandatory for the diagnosis of CAA-ri. Here, we present the case of an 82-year-old man with worsening confusion and bilateral visual loss. MRI revealed no CMBs on T2*, but a small number were detected using thin-slice SWI. Histopathology confirmed Aβ-related angiitis with inflammation and amyloid deposits in the vascular media. Steroid treatment improved clinical outcomes, but mild CMBs and increased cortical superficial siderosis were detected on follow-up MRI. As shown in this case, assessment of CAA-ri requires thorough evaluation of CMBs using SWI because T2* alone may be insufficient. If significant CMBs are minimal, leptomeningeal enhancement may indicate the need for biopsy for early diagnosis.

## Introduction

Cerebral amyloid angiopathy-related inflammation (CAA-ri) is an uncommon but clinically important variant of cerebral amyloid angiopathy (CAA). CAA-ri typically occurs around 73 years of age and has been reported in approximately 10.8% of patients with CAA [1]. It is characterized by autoimmune perivascular inflammation triggered by amyloid-beta (Aβ) deposits in the cortical and leptomeningeal vessels. Because of its overlapping clinical and radiological features with other inflammatory or haemorrhagic disorders, the early diagnosis of CAA-ri remains challenging, and misdiagnosis is not uncommon [1,2]. Definite diagnosis of CAA-ri requires a brain biopsy, but patients with CAA-ri often show a remarkably high number of cerebral microbleeds (CMBs) [3]. Susceptibility-weighted imaging (SWI) is more sensitive than T2* for detecting CMBs, as it incorporates phase information and higher spatial resolution, which can detect smaller or early-stage microbleeds that may be missed on conventional T2* [4,5]. Nevertheless, SWI is not always used for the diagnosis of CAA and CAA-ri [6-8].

Here, we present a case of biopsy-confirmed CAA-ri in a patient who had no CMBs on T2* but had a small number on thin-slice SWI, along with leptomeningeal enhancement.

## Case presentation

An 82-year-old man developed transient visual hallucinations seven days before the current presentation, followed by progressively worsening confusion and bilateral visual loss. Laboratory findings were unremarkable, including inflammatory markers, autoimmune profile, and infectious testing. A cerebrospinal fluid analysis showed elevated protein (210 mg/dL) and IgG index (0.82) with normal white blood cells, cerebrospinal fluid-glucose/serum-glucose ratio, and oligoclonal bands (Table 1). Electroencephalography revealed intermittent focal slowing involving the left temporal to occipital regions, in the absence of epileptiform discharges.

**Table 1:** Laboratory findings and cerebrospinal fluid findings. WBC: white blood cells; CRP: C-reactive protein; BUN: blood urea nitrogen; ANA: antinuclear antibody; c-ANCA: cytoplasmic antineutrophil cytoplasmic antibody; p-ANCA: perinuclear antineutrophil cytoplasmic antibody; ACE: angiotensin-converting enzyme; CSF: cerebrospinal fluid

Test	Result	Reference range
WBC (/μL)	9,100	3,300–8,600
Hemoglobin (g/dL)	10.0	13.7–16.8
Platelet (×10^4^/μL)	18.8	15.8–34.8
CRP (mg/dL)	1.16	0–0.14
Sodium (mEq/L)	141	138–145
Potassium (mEq/L)	2.9	3.6–4.8
BUN (mg/dL)	18.2	8–20
Creatinine (mg/dL)	0.89	0.65–1.07
ANA	Negative	Negative
c-ANCA	Negative	Negative
p-ANCA	Negative	Negative
Beta-D-glucan (pg/mL)	Negative	Negative
ACE (U/mL)	8.9	8.3–21.4
CSF cell count (/μL)	2	0–5
CSF protein (mg/dL)	210	10–40
CSF glucose (mg/dL)	71	50–75
IgG index	0.82	<0.7
Oligoclonal bands	Negative	Negative
CSF culture	Negative	Negative

In relation to the patient’s sight, he had a history of lacunar infarction, hypertension, optic neuritis and angle-closure glaucoma in the left eye, and ischemic optic neuropathy in the right eye. This had resulted in blurred left visual field and stenosis in the right field. However, he had no history of psychosocial or cognitive impairment. Physical examination was unremarkable.

**Figure 1:**
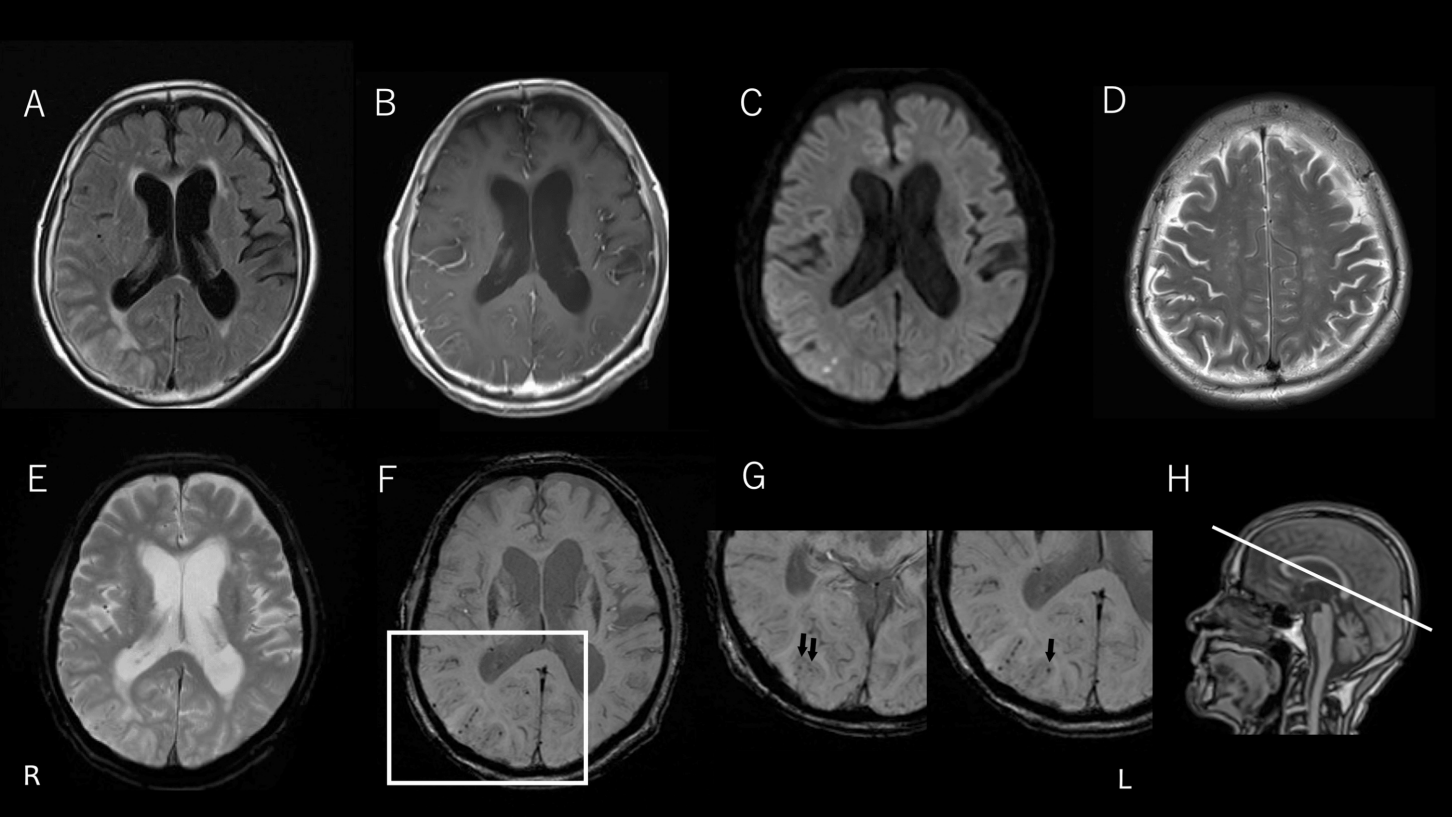
MRI findings at initial presentation. Fluid-attenuated inversion recovery imaging shows multifocal white matter hyperintensities, predominantly in the right occipital lobe (A). Contrast-enhanced T1-weighted imaging reveals leptomeningeal enhancement (B). Diffusion-weighted imaging demonstrates multiple small cortical and subcortical infarcts (C). T2-weighted imaging shows perivascular space dilation in the centrum semiovale (D). No cerebral microbleeds (CMBs) were seen on T2*, although cortical superficial siderosis was noted (E). A small number of CMBs were detectable on thin-slice susceptibility-weighted imaging (F, G).  A sagittal localizer image demonstrated that the T2* and susceptibility-weighted imaging (SWI) sequences were obtained at the same anatomical levels (H).

**Figure 2:**
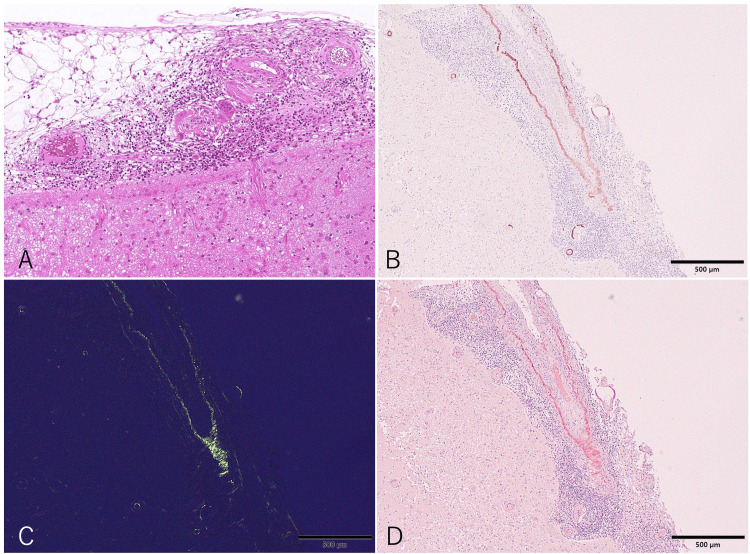
Brain biopsy. Histopathology examination showed perivascular and transmural inflammation with multinucleated giant cells predominantly in the leptomeninges. Hematoxylin-eosin, original magnification 100× (A). Amyloid-beta deposition in the vascular media was demonstrated by Congo red staining (B), Congo red under polarized light (C), and immunohistochemistry (D). Bars = 500 μm.

Considering the combination of visual and cognitive symptoms, we performed an MRI of the brain. It revealed multifocal white matter hyperintensity lesions (leptomeningeal, cortico-subcortical, and deep) predominantly in the right occipital lobe on fluid-attenuated inversion recovery (FLAIR) hyperintensities and T2-weighted imaging. Additionally, contrast-enhanced T1-weighted images showed leptomeningeal enhancement without parenchymal involvement. Diffusion-weighted imaging revealed multiple small scattered infarcts in the cortical and subcortical areas. T2-weighted imaging demonstrated abundant perivascular space dilation in the centrum semiovale. No CMBs were seen on T2*, although slight cortical superficial siderosis (cSS) was detected. However, a detailed examination of the lesion sites using thin-slice SWI uncovered a small number of CMBs (Figure 1). Given the diagnostic uncertainty, a brain biopsy was performed. The biopsy targeted the right occipital cortex and adjacent leptomeninges, corresponding to the region showing the most prominent leptomeningeal enhancement on MRI. Interestingly, histopathological examination revealed predominant infiltration of inflammatory cells in the leptomeninges, accompanied by multinucleated giant cells. Congo red staining and immunohistochemistry demonstrated Aβ deposition in the vascular media. The vessel with Aβ deposition showed a destructive change with transmural inflammation, confirming the diagnosis of CAA-ri (pathologically termed Aβ-related angiitis) (Figure 2).

The patient was started on intravenous methylprednisolone (1 g/day) in three courses, totaling 8 g, followed by a tapering regimen of oral prednisolone (45 mg/day). He gradually became clinically alert and regained some vision over the course of one month. The Mini-Mental State Examination (MMSE) and Hasegawa Dementia Scale-Revised (HDS-R) could not be performed on admission due to confusion. After therapy, vision-independent testing showed 15/25 on the MMSE and 12/25 on the HDS-R. Cerebrospinal fluid analysis normalized, including both protein and IgG index. Follow-up MRI performed one month after treatment initiation showed resolution of previously observed leptomeningeal enhancement on contrast-enhanced T1-weighted images. However, mild CMBs and cSS were detectable, along with changes following the biopsy. The patient was transferred for rehabilitation three months after treatment, with prednisolone tapered to 12.5 mg and modified Rankin Scale 3.

## Discussion

Our patient with CAA-ri had no CMBs on T2*, but a small number of them could be detected on SWI. He showed leptomeningeal enhancement, and then the diagnosis was confirmed by brain biopsy. T2*-weighted MRI alone may be insufficient for this purpose, so we recommend CAA-ri assessment by thorough evaluation of CMBs using thin-slice SWI. When significant CMBs are absent, leptomeningeal enhancement serves as a crucial imaging feature that can be used to examine the possibility of CAA-ri.

The first finding underscores that SWI, not just T2*, is helpful for assessing CMBs in patients with CAA-ri. In CAA-ri, CMBs are a key haemorrhagic lesion that are seen in over 90% of cases, frequently in substantial numbers [2,3]. The revised criteria for CAA-ri reported in a previous study require the presence of at least two of the following haemorrhagic lesions: lobar CMBs, cSS, convexity subarachnoid haemorrhage, lobar intracerebral haemorrhage, and align with the diagnostic principles of the Boston criteria version 2.0 for sporadic CAA [7,8]. Compared with CAA, there are no significant differences in chronic intracerebral haemorrhage or cSS, highlighting the diagnostic relevance of CMBs in CAA-ri [3]. Our patient exhibited only minimal cSS on both T2* and SWI without any evidence of CMBs on T2*. A detailed analysis of the lesion areas on thin-slice SWI revealed a small number of CMBs. SWI is known to have higher sensitivity and reliability for quantifying the CMB counts than T2* images. Studies have shown that it detects approximately two to three times more CMBs [2,4,8]. However, in some currently proposed diagnostic criteria for CAA-ri, SWI is not required and can be substituted with T2* [6-8]. Patients with probable CAA-ri detected using SWI might therefore be reclassified as undetermined CAA-ri based on T2*, as seen in our case. CMBs may not be detected on T2*, but a careful inspection of the lesion sites with thin-slice SWI can reveal a small number of CMBs, which could lead to a suspected diagnosis of CAA-ri.

The second finding highlights the diagnostic significance of leptomeningeal enhancement in CAA-ri with minimal microbleeds. In our patient’s case, leptomeningeal enhancement was shown, which has been reported in 70% of cases of CAA-ri [2]. The proposed CAA-ri diagnostic criteria have been revised to incorporate leptomeningeal markers in an attempt to avoid underdiagnosis and misdiagnoses [7,9]. Pathologically, contrast enhancement, especially leptomeningeal enhancement, is twice as common in transmural inflammation (Aβ-related angiitis) compared to perivascular inflammation in CAA-ri [10]. Our case also confirmed the pathological findings of vasculitis targeting Aβ-positive vessels. Similar to our case, a small number of case reports have described follow-up MRI revealing microbleeds that were not initially detected. There is the suggestion that they may develop secondary to increased leptomeningeal vascular inflammation [9,11]. Early immunosuppressive treatment in this case led to significant clinical and radiological improvement, which highlights the importance of early diagnosis and treatment [12]. Leptomeningeal enhancement may therefore play an important role in the early detection and management of CAA-ri. Although brain biopsy can contribute to a definitive diagnosis, its potential haemorrhagic risks should be carefully considered in patients with amyloid angiopathy, with evaluation of imaging and genetic factors, such as cSS and the apolipoprotein E ε2 allele [13,14].

## Conclusions

We described a case of biopsy-confirmed CAA-ri with the absence of CMBs on T2* and detectable only by SWI. The assessment of CAA-ri may be aided by a comprehensive evaluation of CMBs using SWI because T2* alone may be inadequate. In cases where CMBs are minimal, leptomeningeal enhancement serves as a crucial imaging feature and may warrant biopsy for early diagnosis.
